# Predictive Prognosis Value of CRP Measurement and CAR in Dogs Infected with Parvovirus

**DOI:** 10.3390/vetsci12121126

**Published:** 2025-11-27

**Authors:** Miguel M. Maximino, Diana O. Lopes, Luísa Mateus, Salomé Gonçalves, Pâmela Valente, Telmo Nunes, Luís M. Tavares, Virgílio S. Almeida, Nuno Sepúlveda, Solange A. Gil

**Affiliations:** 1 Faculty of Veterinary Medicine, University of Lisbon, Av. Universidade Técnica, 1300-477 Lisbon, Portugal; mmaximino@fmv.ulisboa.pt (M.M.M.); dianalopes@fmv.ulisboa.pt (D.O.L.); lmateus@fmv.ulisboa.pt (L.M.); tnunes@fmv.ulisboa.pt (T.N.); ltavares@fmv.ulisboa.pt (L.M.T.); vsa@fmv.ulisboa.pt (V.S.A.); 2 CIISA—Centre of Interdisciplinary Research in Animal Health, Faculty of Veterinary Medicine, University of Lisbon, Av. Universidade Técnica, 1300-477 Lisbon, Portugal; pamelavalente@fmv.ulisboa.pt; 3 Associate Laboratory for Animal and Veterinary Sciences (AL4AnimalS), 1300-477 Lisbon, Portugal; 4 Teaching Hospital, Faculty of Veterinary Medicine, University of Lisbon, Av. Universidade Técnica, 1300-477 Lisbon, Portugal; salome@fmv.ulisboa.pt; 5 Faculty of Mathematics and Information Science, Warsaw University of Technology, Koszykowa 75, 00-637 Warsaw, Poland; nuno.sepulveda@pw.edu.pl; 6 CEAUL—Centro de Estatística e Aplicações da Universidade de Lisboa, 1749-016 Lisbon, Portugal

**Keywords:** biomarkers, C-reactive protein, C-reactive protein/albumin ratio, dogs, systemic inflammatory response syndrome, parvovirus, veterinary medicine

## Abstract

Canine parvoviral (CPV) enteritis is frequently associated with systemic inflammatory response syndrome (SIRS), yet objective biomarkers for early detection remain limited. This study evaluated the diagnostic performance of C-reactive protein (CRP), albumin, and the CRP/albumin ratio (CAR) in 60 dogs (45 CPV-positive and 15 healthy controls). CRP and CAR were markedly higher in infected dogs, while albumin was significantly reduced (all *p* < 0.001). ROC analysis demonstrated excellent discrimination for SIRS, with AUC values of 0.87 for CRP and 0.86 for CAR and optimal ROC01 thresholds of 2.25 mg/dL and 1.23, respectively. The IDEXX CRP-based SIRS classification showed moderate agreement with the clinical reference (κ = 0.56), whereas a conditional inference tree and logistic regression models confirmed CRP as the most informative variable. The final age-adjusted model including the interaction age × CRP achieved superior performance (AUC = 0.93; κ = 0.84), indicating that CRP’s predictive effect varied with age. These results demonstrate that CRP and CAR are complementary, accessible biomarkers for assessing systemic inflammation in CPV infection and suggest that the IDEXX CRP cut-off (>3 mg/dL) may be conservative, as values above 2.25 mg/dL already reflect clinically relevant inflammatory activity.

## Simple Summary

Canine parvoviral enteritis often triggers systemic inflammatory response syndrome (SIRS), which can complicate clinical management and worsen prognosis. We examined the usefulness of two inflammatory serum markers, C-reactive protein (CRP) and the CRP/albumin ratio (CAR), in a population of dogs with naturally acquired parvoviral infection. Both CRP and CAR were significantly higher in infected dogs compared with healthy controls, and their concentrations were strongly associated with the presence of SIRS. The optimal diagnostic thresholds obtained from ROC analysis were slightly lower than the manufacturer’s reference limits, suggesting that dogs within the “mild or resolving inflammation” CRP range (1–3 mg/dL) may already exhibit systemic inflammation. Logistic regression identified an interaction between age and CRP, highlighting the need to consider age effects when interpreting acute-phase responses. These findings support the combined use of CRP and CAR as accessible, clinically meaningful indicators for early detection and monitoring of systemic inflammation in canine parvoviral infection.

## 1. Introduction

Biomarkers are measurable indicators of physiological and pathological processes that provide valuable insight into disease mechanisms, early diagnosis, therapeutic monitoring, and prognosis in both human and veterinary medicine [1,2]. Among these, acute phase proteins (APPs) are a major class of biomarkers mirroring systemic inflammatory activity [3,4,5]. The acute phase response (APR) represents an early and non-specific systemic reaction to infection, tissue injury, or inflammation, aiming to re-establish homeostasis through metabolic and immune adjustments [6,7]. During APR, hepatic synthesis of APPs is either up-regulated (positive APPs) or down-regulated (negative APPs) in proportion to the intensity of the inflammatory stimulus, making these proteins reliable surrogates for disease severity [6,8].

C-reactive protein (CRP) is one of the most extensively studied positive APPs in veterinary medicine. It rises rapidly, within hours, following inflammatory stimulation and decreases promptly once the underlying cause resolves, making it a sensitive biomarker for real-time monitoring of disease activity [9,10]. Raised CRP concentrations have been associated with several canine diseases involving systemic inflammation, such as sepsis, bacterial pneumonia, and systemic inflammatory response syndrome (SIRS) [11,12,13]. Although the magnitude of increase varies by etiology and disease severity, CRP consistently correlates with clinical deterioration, response to therapy, and outcome. Conversely, albumin, a major negative APP, typically decreases during inflammation due to hepatic reprioritization of protein synthesis, increased vascular permeability, and redistribution mechanisms [9].

The ratio between these two proteins, the C-reactive protein to albumin ratio (CAR), recently emerged as an integrated index of systemic inflammation and disease severity [14,15]. By combining a positive and a negative APP, CAR reflects both the inflammatory and nutritional/metabolic components of the APR. In human medicine, CAR has shown strong diagnostic and prognostic value in sepsis, cancer, and critical illness, often outperforming CRP or albumin alone in predicting outcomes [16]. In veterinary medicine, its application is still limited, but early evidence suggests that CAR may better capture the overall inflammatory burden and predict mortality more accurately than CRP alone in dogs with severe systemic disease [17].

SIRS represents a widespread inflammatory state that can result from both infectious and non-infectious stimuli [18]. Its clinical recognition in veterinary practice remains challenging because diagnostic criteria (temperature, heart rate, respiratory rate, and leukocyte count) are relatively non-specific and may be influenced by stress, dehydration, or therapeutic interventions [19]. Consequently, objective biomarkers such as CRP and CAR may complement clinical evaluation, offering quantitative support for identifying systemic inflammation and guiding early therapeutic decisions.

Canine parvoviral enteritis (CPV) is a severe, highly contagious viral disease that provides a natural model of acute systemic inflammation [20,21]. Viral replication in intestinal crypt cells causes epithelial destruction and mucosal barrier loss, facilitating bacterial translocation and endotoxemia, which can precipitate SIRS and sepsis [22,23]. The degree of systemic inflammation in CPV varies widely and is strongly correlated with clinical severity, prognosis, and mortality [11]. Consequently, this infectious disease offers a unique framework for evaluating the diagnostic and prognostic performance of inflammatory biomarkers such as CRP and CAR under naturally occurring systemic inflammation.

Therefore, this study aimed to evaluate the diagnostic performance of CRP, albumin, and CAR in dogs naturally infected with canine parvovirus, with emphasis on their association with SIRS. Beyond comparing these biomarkers between clinical groups, we sought to determine their optimal diagnostic thresholds and agreement with the IDEXX CRP-based classification. In addition, age-adjusted logistic regression models and decision tree analyses were used to identify the most explanatory predictors of SIRS and to assess the potential interaction between demographic and biochemical variables. By integrating clinical, biochemical, and statistical modelling approaches, this study aimed to clarify the utility of CRP and CAR as accessible, objective indicators of systemic inflammation in canine parvoviral infection.

## 2. Materials and Methods

### 2.1. Study Population

Serum specimens were obtained from forty-five dogs naturally infected with CPV during their hospitalization in the Biological Isolation and Containment Unit (BICU) of the Veterinary Teaching Hospital (VTH), Faculty of Veterinary Medicine, University of Lisbon (FMV-ULisboa).

Additionally, fifteen serum samples from clinically healthy dogs presented for elective surgery (e.g., neutering) or routine vaccination at the same hospital were used as controls. According to clinical status, two groups were defined: the Parvovirus Group (PG) and the Control Group (CG). It was not ethically permissible to obtain blood samples from very young, clinically healthy puppies solely for research purposes. Therefore, healthy controls were recruited only among dogs already undergoing venipuncture for clinical procedures (e.g., vaccination, pre-anesthetic testing). As these animals are typically older than the parvovirus-infected population, age-matching between groups was not feasible. This design constraint explains the age imbalance between PG and CG and reinforces the need to adjust for age in all subsequent analyses. All procedures were conducted by the same medical team under standardized clinical protocols and biosecurity guidelines. Written informed consent for the research use of clinical data was obtained from dog owners at the time of each appointment or hospitalization. All data handling and analysis occurred only after the study received ethical clearance from the Ethics Committee of the Faculty of Veterinary Medicine, University of Lisbon (Protocol 024/2023).

### 2.2. Inclusion and Exclusion Criteria

The PG included dogs with confirmed parvoviral infection by real-time polymerase chain reaction (RT-qPCR) performed on fecal and/or blood samples. When RT-qPCR was unavailable, diagnosis was based on a positive immunochromatographic test, provided that the dogs had not been vaccinated or that more than three weeks had elapsed since the last vaccination. Blood samples for biochemical analysis were collected within 24 to 48 h after hospitalization to determine CRP concentrations. Only a single CRP measurement per animal was considered, recognizing that initial supportive therapy had already been instituted upon admission. Eligible dogs were younger than three years of age and hospitalized for at least three days, ensuring that both clinical reassessment and repeat analytical testing could be performed. Inclusion also required availability of an admission hemogram and a second hemogram obtained between 48 and 72 h after hospitalization.

The CG comprised clinically healthy dogs aged three years or younger without evidence of systemic or inflammatory disease on physical examination and with complete hematologic and biochemical evaluations performed during routine consultation for elective procedures or vaccination.

Dogs that did not fulfill all inclusion criteria for either group or that presented incomplete clinical or analytical records were excluded from the study.

### 2.3. Sample Collection, Storage, Analysis and Testing

Blood samples (2 mL) were collected by jugular or saphenous venipuncture into plain tubes for serum separation. Samples were centrifuged at 5000× *g* for 10 min, and the serum was immediately stored at −80 °C until biochemical analysis.

CRP concentrations were determined using CRP IDEXX Catalyst^TM^ on a IDEXX Catalyst One^TM^ Analyzer (IDEXX Laboratories, Inc., Westbrook, ME, USA) at the Braço Forte Clinical Analysis Laboratory, FVM-ULisboa. The analyzer provided both quantitative CRP values and qualitative classifications, according to the value of CRP measured (see Section 2.5). Serum albumin was measured by bromocresol-green colorimetry using the Albumin FS kit following the analyzer’s internal protocol (Response^®^ 910 Vet, DiaSys, Wixom, MI, USA).

Blood samples preserved with EDTA were used to run complete blood counts (CBCs). Hematological analyses were conducted using automated analyzers, specifically, the IDEXX ProCyte One^®^ (IDEXX Laboratories, Inc., Westbrook, ME, USA) and the Zoetis VETSCAN^®^ HM5 (Zoetis, Parsippany-Troy Hill, NJ, USA).

Detection of CPV infection was performed at the Virology and Immunology Laboratory (VIL) of the FVM-ULisboa. When available, RT-qPCR was used for confirmation on fecal and/or blood samples. In cases where RT-qPCR was not feasible, diagnosis relied on the WITNESS^®^ Parvo rapid immunomigration test (Zoetis Inc., USA), which has a specificity of 96.1% with a 95% confidence interval (95% CI) of 89.2–98.7% and a sensitivity of 86.3% (95% CI: 76.6–92.4%) [24], thereby providing a reliable alternative diagnostic method. Among the forty-five dogs in the parvovirus group, 40 (88.9%) were diagnosed by RT-qPCR performed on fecal and/or blood samples, while 5 dogs (11.1%) were diagnosed using the WITNESS^®^ Parvo immunochromatographic test.

### 2.4. Clinical Variables

Clinical, hematological, and biochemical parameters were recorded within the first 24 to 48 h of hospitalization for all dogs in the PG to characterize each animal’s physiological and inflammatory condition.

Data collected included breed (purebred or mixed-breed), sex (male or female), age (in months), duration of hospitalization (HT) in days, and clinical outcome (discharge or death). Vital signs documented during physical examination comprised heart rate (HR), respiratory rate (RR), and rectal temperature (°C).

Hematological parameters, such as total leukocyte count (TLC) were obtained from the CBC performed during this period.

Biochemical parameters included serum albumin and CRP concentrations, both measured from samples collected within 24–48 h after admission. Albumin values were also re-evaluated 48 h after hospitalization to assess hypoalbuminemia trends. The CAR was calculated for each animal as CRP divided by albumin and subsequently evaluated as a composite marker of inflammation.

### 2.5. SIRS Classification

Two complementary strategies were employed to classify animals as being in SIRS or not.

The first classification, considered the clinical reference standard, followed the criteria proposed by Sykes [19], which evaluate four physiological parameters: body temperature, HR, RR, and TLC. A dog was classified as SIRS-positive when at least two of these parameters were outside the normal reference range, indicating the presence of clinically relevant systemic inflammation (See Supplementary Materials, Table S1).

The second classification relied on CRP concentrations obtained from the IDEXX Catalyst Analyzer within the same 24–48 h time frame. According to the manufacturer’s reference ranges, dogs with a CRP < 1.0 mg/dL were considered unlikely to have systemic inflammation, values between 1.0 and 3.0 mg/dL were interpreted as mild or resolving inflammation, and a CRP > 3.0 mg/dL indicated a severe systemic inflammatory process [25]. For statistical purposes, and to maintain binary comparability with the Sykes classification, animals with CRP values between 1.0 and 3.0 mg/dL were categorized as SIRS-negative.

### 2.6. Statistical Analysis

All data were organized in Microsoft^®^ Excel^®^ 2019 (Microsoft Corporation, Redmond, WA, USA) for dataset management and descriptive summaries. Statistical analyses were conducted on a MacBook Pro (Apple Inc., Cupertino, CA, USA) running macOS Tahoe 26.1 (M1 Pro processor, 16 GB RAM) using R software (version 4.2.2; R Foundation for Statistical Computing, Vienna, Austria). Statistical significance was set at *p* < 0.05.

Quantitative variables were tested for normality using the Shapiro–Wilk test. Since most continuous variables exhibited non-normal distributions (*p* < 0.05), data are reported as medians and interquartile ranges (IQRs), and non-parametric methods were used for group comparisons. To assess differences between PG and CG, continuous variables were analyzed with the Wilcoxon rank-sum (Mann–Whitney U) test, whereas categorical variables were evaluated using Fisher’s exact test.

Within the total population, empirical cumulative distribution function (ECDF) plots were generated to visualize the distribution of CRP, albumin, and CAR across clinical groups. The relationships between CRP, albumin, CAR, and age were assessed using Spearman’s rank correlation coefficient (ρ).

Comparisons of CRP, albumin, and CAR levels between SIRS-positive and SIRS-negative dogs (according to Sykes’ criteria) were conducted using the Wilcoxon rank-sum test.

Agreement between the clinical SIRS classification (Sykes) and the IDEXX-based CRP classification was evaluated using Cohen’s kappa (κ) coefficient, and diagnostic performance indices, including sensitivity, specificity, positive predictive value (PPV), negative predictive value (NPV), accuracy, and 95% confidence intervals (CIs), were computed using the *epiR* package (version 2.0.88) [26].

To investigate potential classification thresholds, a conditional inference decision tree was constructed (using the *partykit* package, version 1.2-20) [27] with CRP, albumin, CAR, and age as predictors of SIRS status. The optimal CRP cut-off identified by the tree was compared with ROC-derived thresholds and with the IDEXX reference criterion.

To model the probability of SIRS, multiple logistic regression models were fitted sequentially following a manual stepwise approach. The models included (1) age alone; (2) age plus each biomarker (CRP, albumin, or CAR); and (3) interaction terms (age × CRP). The model with the lowest Akaike Information Criterion (AIC) was selected as the final model. Odds ratios (ORs) with 95% CIs were calculated, and model discrimination was evaluated by the area under the curve (AUC), sensitivity, specificity, PPV, NPV, accuracy, and Cohen’s κ.

Receiver operating characteristic (ROC) analyses were performed using the *pROC* package (version 1.19.0.1) [28] to assess the discriminative performance of individual biomarkers (CRP, albumin, and CAR) and of the logistic regression models. The AUC and corresponding 95% CIs were computed using DeLong’s method. Optimal cut-off values were determined according to the closest-to-(0,1) criterion (ROC01) and visualized on the ROC plots.

## 3. Results

### 3.1. Study Population and Demographic Characterization

A total of 60 dogs were included in the study, comprising 45 parvovirus-infected cases (PG) and 15 healthy controls (CG) as described in Table 1.

Parvovirus-infected dogs were significantly younger than controls (median age 2.0 [2.0–4.0] months compared 12.0 [11.0–12.0]; *p* < 0.001), reflecting that the groups were not age-matched.

No significant differences were observed in sex or breed distribution between the groups (*p* = 0.084 and 0.192, respectively).

Serum CRP and the CRP/albumin ratio (CAR) were markedly higher in parvovirus-infected dogs compared with controls (both *p* < 0.001), while albumin levels were significantly lower (*p* < 0.001). Leukocyte count and body temperature did not differ significantly between groups (both *p* > 0.35).

**Figure 1:**
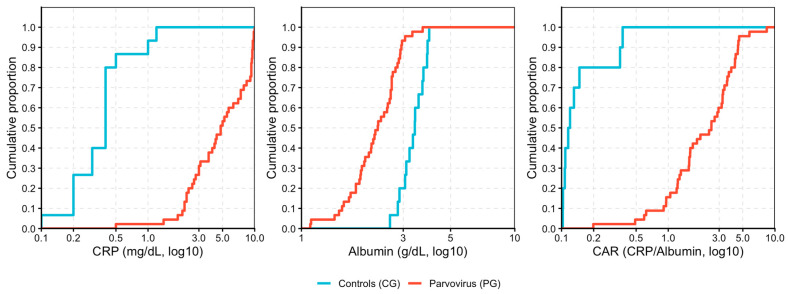
Empirical cumulative distribution function (ECDF) plots for log-transformed CRP, albumin, and CRP/albumin ratio (CAR) in parvovirus-infected dogs (PG, red) and healthy controls (CG, blue).

Figure 1 displays the empirical cumulative distribution function (ECDF) plots for log10-transformed CRP, albumin, and CAR. The cumulative curves highlight a clear and consistent separation between parvovirus-infected dogs (PG) and healthy controls (CG) across the entire value range. For CRP and CAR, the PG curve shifts markedly to the right, indicating uniformly higher concentrations among infected dogs. Conversely, for albumin, the PG curve is displaced to the left, reflecting a generalized reduction in serum levels.

### 3.2. CRP and Albumin Analysis in Relation to SIRS (Sykes)

CRP and albumin concentrations showed opposite trends in relation to SIRS classification (Sykes’ criteria).

Median CRP was markedly higher in SIRS-positive dogs (4.8 [2.8–9.1] mg/dL) compared with SIRS-negative animals, which exhibited markedly lower values (0.4 [0.25–0.40] mg/dL; *p* < 0.001).

In contrast, serum albumin was significantly reduced in SIRS-positive dogs (2.28 [1.89–2.65] g/dL) compared with SIRS-negative animals (3.40 [2.80–3.70] g/dL; *p* = 0.001) (See Supplementary Materials Table S2 for statistical analysis).

### 3.3. Correlation Analysis

Spearman’s rank correlation coefficients between CRP, albumin, CAR, and age are presented in Table 2.

CRP was moderately and negatively correlated with albumin (ρ = −0.53, *p* < 0.001) and age (ρ = −0.38, *p* = 0.003), and strongly and positively correlated with CAR (ρ = 0.96, *p* < 0.001).

Albumin showed a strong negative correlation with CAR (ρ = −0.70, *p* < 0.001) and a positive correlation with age (ρ = 0.54, *p* < 0.001). CAR was moderately and negatively correlated with age (ρ = −0.43, *p* < 0.001).

### 3.4. CAR and SIRS Association

CAR was significantly higher in SIRS-positive dogs compared with SIRS-negative dogs (2.54 [1.28–3.58] and 0.11 [0.07–0.13], respectively; *p* < 0.001); see Supplementary Materials Table S2 for Wilcoxon rank-sum test results.

### 3.5. Agreement Between CRP-Based and Clinical SIRS Classifications

The IDEXX CRP-based SIRS classification showed a statistically significant association with the clinical reference (Sykes’ criteria) and moderate agreement between methods (Cohen’s κ = 0.56). Diagnostic performance metrics for the IDEXX system, including sensitivity, specificity, and predictive values, are summarized in Table 3.

### 3.6. ROC Curve Analysis for CRP and CAR

**Figure 2:**
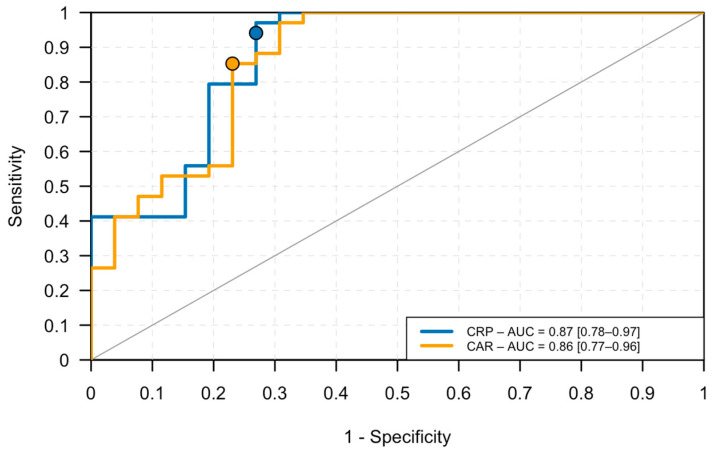
ROC curves for CRP and CAR in predicting SIRS (Sykes’ criteria). The solid dots on each curve represent the ROC01 optimal cut-off points (closest-to-(0,1) criterion).

As illustrated in Figure 2, ROC analysis using SIRS (Sykes) as the reference standard demonstrated excellent discriminative ability for both CRP (AUC = 0.87, 95% CI: 0.78–0.97) and CAR (AUC = 0.86, 95% CI: 0.77–0.96).

The optimal cut-off points, determined using the ROC01 (closest-to-(0,1)) criterion, were 2.25 mg/dL for CRP (sensitivity = 0.94; specificity = 0.73) and 1.23 for CAR (sensitivity = 0.85; specificity = 0.77).

### 3.7. Decision Tree Results

**Figure 3:**
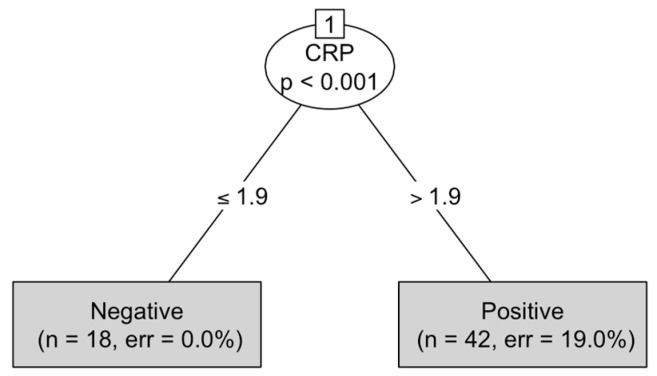
Conditional inference decision tree identifying predictors of systemic inflammatory response syndrome (SIRS) in dogs naturally infected with canine parvovirus.

A conditional inference decision tree including age, CRP, albumin, and CAR as predictors identified CRP as the sole significant variable associated with SIRS (Sykes’ criteria), as illustrated in Figure 3.

The model established an optimal split at 1.9 mg/dL (*p* < 0.001), classifying dogs with CRP ≤ 1.9 mg/dL as SIRS-negative and those with CRP > 1.9 mg/dL as SIRS-positive. The resulting tree achieved an overall classification accuracy of 0.87, sensitivity = 0.69 (95% CI: 0.48–0.86), specificity = 1.00 (95% CI: 0.90–1.00), and Cohen’s κ = 0.72. (See Supplementary Tables S3 and S4 for full model performance). Node counts in the decision tree reflect the model-based reclassification according to the CRP split at 1.9 mg/dL and do not correspond to the true clinical SIRS status of the animals. As the tree uses CRP as the only predictor, some healthy controls with mildly increased CRP values were classified as ‘Positive’ by the model, despite not being clinically SIRS-positive.

### 3.8. Logistic Regression Analysis

Because study groups were not age-matched, age was included as a covariate in all logistic regression models. A manual stepwise regression approach was applied to identify the most parsimonious model for predicting SIRS status (Sykes’ criteria), sequentially testing age, CRP, albumin, and CAR.

The age-only model (model 1) showed a significant negative association with SIRS (*p* < 0.001; AIC = 62.97). Adding CRP (model 2a) markedly improved the model fit (AIC = 49.14), with both age (*p* = 0.003) and CRP (*p* = 0.002) remaining significant. Models including albumin (model 2b) or CAR (model 2c) yielded higher AICs (63.56 and 52.45, respectively). Adding these variables to the age + CRP model (models 3a–3b) did not improve performance (AICs = 51.10 and 50.84).

The best-fitting model was obtained when the interaction term between age and CRP was included (model 4, AIC = 43.82). In this model, age (*p* = 0.019) and the interaction term age × CRP (*p* = 0.049) were significant, indicating that the effect of CRP on SIRS probability was age-dependent (see Supplementary Tables S5 and S6 for detailed model coefficients and performance metrics).

**Figure 4:**
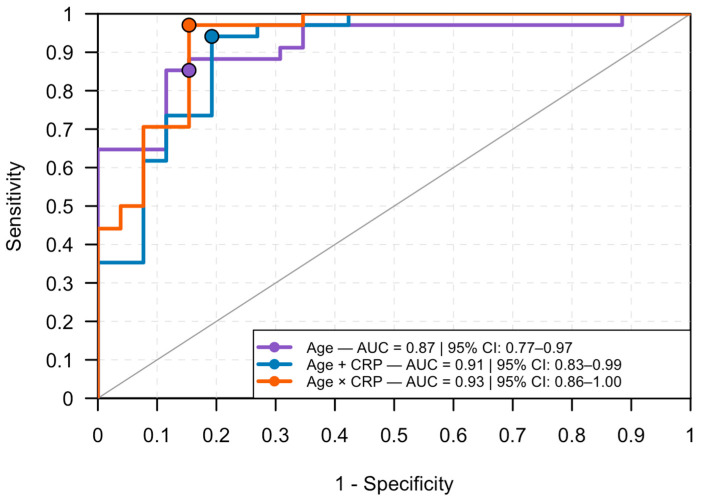
Receiver operating characteristic (ROC) curves for logistic regression models predicting systemic inflammatory response syndrome (SIRS) in dogs naturally infected with canine parvovirus. The solid dots on each curve represent the ROC01 optimal cut-off points (closest-to-(0,1) criterion).

Receiver operating characteristic (ROC) analysis confirmed progressive improvement across models: age alone (AUC = 0.87, 95% CI: 0.77–0.97), age + CRP (AUC = 0.91, 95% CI: 0.83–0.99), and the final age × CRP model (AUC = 0.93, 95% CI: 0.86–1.00), demonstrating excellent discriminative capacity (Figure 4).

At the optimal probability threshold (ROC01 = 0.69), the age × CRP model achieved a PPV 0.97, and NPV 0.85. Cohen’s κ = 0.84 (95% CI: 0.69–0.99) (See Supplementary Table S6).

## 4. Discussion

### 4.1. Demographic and Baseline Characteristics

The present study included 60 dogs, of which, 45 were naturally infected with CPV and 15 served as healthy controls. As expected for this infection, parvovirus-positive dogs were significantly younger than controls (median age 2 months vs. 12 months, *p* < 0.001), confirming the well-recognized increased susceptibility of juvenile dogs to CPV enteritis [23,29,30]. This age imbalance reflects the epidemiological profile of CPV, which predominantly affects puppies during the post-weaning period, when maternal antibodies decline and active immune competence is still developing [31]. The immature intestinal epithelium and rapid cell turnover in young dogs further facilitate viral replication and systemic dissemination, predisposing to severe systemic inflammatory responses [32].

No significant differences in sex or breed distribution were detected between groups, suggesting that these factors did not contribute to the observed variation in biomarker levels. Nevertheless, because the groups were not age-matched, age was included as a covariate in all regression models to account for its potential confounding or modifying effects.

### 4.2. Overview of CRP, Albumin, and CAR as Biomarkers of Systemic Inflammation

Both CRP and CAR were markedly elevated in affected dogs compared with healthy controls, while albumin concentrations were significantly reduced, collectively confirming a robust acute-phase reaction consistent with severe systemic inflammation [33].

Figure 1 displays the ECDF plots for log-transformed CRP, albumin, and CAR. These non-overlapping distribution patterns visually confirm the strong statistical contrasts observed in the Wilcoxon tests and illustrate that inflammatory activation in CPV infection is not limited to a subset of animals but affects the population as a whole.

CRP is a well-established major acute-phase protein in canine medicine, showing rapid induction within hours of inflammatory stimulation and a short plasma half-life that enables real-time monitoring of disease activity [33,34,35,36]. The concentrations observed in this study (median 4.8 mg/dL; IQR 2.8–9.1) fall within the ranges previously reported for CPV enteritis, sepsis, and severe bacterial infections [11,12,37,38]. In parallel, the decrease in albumin among parvovirus-infected and SIRS-positive dogs aligns with its role as a negative acute-phase protein, downregulated under pro-inflammatory cytokine signaling and redistributed due to vascular permeability changes [2,39].

Beyond group medians, the upper tail of the CRP distribution provides additional clinical insight. Approximately one-quarter of the dogs (26.7%) exhibited markedly elevated CRP concentrations (>9.0 mg/dL), consistent with reports from tertiary-care hospitals managing severe systemic disease [40]. Such extreme values, rarely found in general practice, are expected in high-acuity populations such as CPV-infected dogs and reflect the magnitude of systemic inflammation and tissue injury. Notably, dogs with the highest CRP levels also displayed the highest CAR values and the lowest albumin concentrations, illustrating the ratio’s ability to highlight the interplay between hyper- and hypo-reactive acute-phase proteins. Similar combined responses have been described in canine pancreatitis [41] and in human septic shock [42,43], where elevated CAR parallels cytokine surges, vascular leakage, and endothelial dysfunction.

By integrating these two proteins with opposing kinetic patterns, CAR provides a more comprehensive reflection of the inflammatory and metabolic burden. The elevated CAR levels found in this cohort (median = 2.54 in SIRS-positive dogs vs. 0.11 in SIRS-negative; *p* < 0.001) therefore reinforces its potential as a clinically valuable indicator of systemic inflammation and metabolic compromise in canine parvoviral infection.

### 4.3. Relationships Among CRP, Albumin, CAR, and SIRS Classification

CRP and albumin showed opposite patterns in relation to the clinical classification of SIRS, as defined by Sykes’ criteria [19]. Median CRP concentrations were higher in SIRS-positive dogs compared with SIRS-negative animals (4.8 mg/dL and 0.4 mg/dL, respectively; *p* < 0.001), whereas albumin levels were significantly lower (2.28 g/dL and 3.40 g/dL, respectively; *p* = 0.001). These inverse trends illustrate the typical behavior of acute-phase proteins during systemic inflammation, CRP acting as a positive acute-phase driven by interleukin-6, and albumin as a negative acute-phase protein suppressed by hepatic reprioritization of protein synthesis [2,39]. CAR magnified this divergence, showing a difference between SIRS-positive and SIRS-negative dogs (2.54 and 0.11, respectively; *p* < 0.001).

The correlation matrix further supports the interplay between these biomarkers. CRP correlated moderately and negatively with albumin (ρ = −0.53, *p* < 0.001) and strongly and positively with CAR (ρ = 0.96, *p* < 0.001). Albumin also showed a strong inverse correlation with CAR (ρ = −0.70, *p* < 0.001). Although CRP and albumin are physiologically linked through the acute-phase response, their only moderate correlation indicates that they capture partially distinct aspects of systemic inflammation. This partial independence supports the usage of CAR, as it integrates the dynamic behavior of both markers. The strong associations between CAR, CRP, and albumin are expected given that CAR is mathematically derived from these components, yet the ratio’s clinical value lies in amplifying their divergence and stabilizing transient fluctuations observed in single biomarkers.

Both CRP and CAR were inversely associated with age, whereas albumin increased with age (ρ = 0.54, *p* < 0.001). Considering that the groups were not age-matched, these findings emphasize the importance of adjusting for age effects in subsequent models.

### 4.4. Agreement Between SIRS Classifications

Comparison between the Sykes clinical criteria [19] and the IDEXX CRP-based reference [25] revealed moderate agreement (Cohen’s κ = 0.56), indicating partial but meaningful overlap between physiological and biomarker-based definitions of systemic inflammation. This level of agreement is consistent with the notion that each method captures distinct yet complementary aspects of the systemic inflammatory response.

The Sykes classification depends on dynamic physiological parameters, HR, RR, temperature, and TLC, that can fluctuate with hydration status, stress, analgesia, or treatment initiation [19,23]. In contrast, the IDEXX classification relies exclusively on serum CRP concentration as an objective biochemical index of inflammation. Similar inconsistencies have been described in human sepsis frameworks, where the Society of Critical Care Medicine and Infectious Diseases Society of America have acknowledged that clinical SIRS criteria exhibit limited discriminant and concurrent validity. They may be present in many hospitalized patients without infection or adverse outcomes, while some septic cases with organ dysfunction may not meet classic SIRS criteria [18]. Biomarker-based definitions, such as CRP, offer greater objectivity but remain constrained by delayed kinetics, variable specificity, and the absence of universally accepted thresholds [44]. Furthermore, dogs in this study received supportive therapy immediately upon hospitalization, which may have modified physiological parameters by the time of evaluation [41,45].

### 4.5. Diagnostic Performance of CRP and CAR (ROC Curve Analysis)

ROC curve analysis demonstrated excellent discriminative performance for both CRP and CAR in identifying dogs that fulfilled the clinical criteria for SIRS. The AUC was 0.87 for CRP (95% CI: 0.78–0.97) and 0.86 for CAR (95% CI: 0.77–0.96), confirming that both biomarkers achieved high accuracy in distinguishing dogs with systemic inflammation.

Optimal cut-off points were identified using ROC01, indicating a CRP threshold of 2.25 mg/dL (sensitivity = 0.94; specificity = 0.73) and a CAR threshold of 1.23 (sensitivity = 0.85; specificity = 0.77). These values suggest that CRP maximizes sensitivity, making it an effective screening marker, whereas CAR achieves a slightly more balanced profile between sensitivity and specificity.

Particularly, the ROC-derived CRP threshold of 2.25 mg/dL was slightly lower than the IDEXX Catalyst^®^ interpretative reference [25], which classifies CRP concentrations 1.0–3.0 mg/dL as “mild or resolving inflammation” and >3.0 mg/dL as consistent with SIRS. In this study, values within the 1.0–3.0 mg/dL range were categorized as negative to maintain a binary classification framework. However, the proximity between our optimal ROC threshold (2.25 mg/dL) and the lower boundary of the IDEXX positive range suggests that dogs with CRP values between 2 and 3 mg/dL may already show biochemical evidence of systemic inflammation. This observation indicates that the manufacturer’s current interpretative threshold may be conservative and that dogs within the “unlikely to have systemic inflammation” range could represent an early or subclinical stage of SIRS development.

### 4.6. Decision Tree Analysis

The conditional inference decision tree provided a complementary, model-free approach to evaluate the predictive performance and clinical interpretability of the studied biomarkers. Among all candidate variables (CRP, albumin, CAR, and age), CRP emerged as the only statistically significant predictor of SIRS classification (*p* < 0.001), highlighting its central role in the systemic inflammatory response associated with parvoviral infection The model established an optimal split at 1.9 mg/dL, effectively distinguishing dogs classified as SIRS-negative from those classified as SIRS-positive.

This threshold closely approximated the value identified through ROC analysis (2.25 mg/dL) and fell below the IDEXX Catalyst^®^ positive criterion (>3.0 mg/dL). Taken together, these findings suggest that dogs with CRP concentrations between 1.0 and 3.0 mg/dL, currently considered “unlikely to have systemic inflammation” by the manufacturer, may already display biochemical evidence of systemic inflammation. The decision tree therefore reinforces that the IDEXX interpretative threshold could be conservative and that mild CRP elevations below 3.0 mg/dL might represent the early phase of SIRS activation.

The resulting model achieved an overall classification accuracy of 0.87, with a sensitivity of 0.69 (95% CI 0.48–0.86), specificity of 1.00 (95% CI 0.90–1.00), and Cohen’s κ = 0.72, indicating substantial agreement with the clinical classification.

### 4.7. Multivariable Logistic Regression Models

Because the study groups were not age-matched, age was included as a covariate in all logistic regression analyses to account for potential confounding effects. Inflammatory markers, particularly CRP, are influenced by physiological maturation, immune competence, and hepatic protein synthesis, all of which differ substantially between puppies and adult dogs [46]. Adjusting for age was therefore essential to ensure that observed associations with SIRS reflected inflammatory processes rather than age-related physiological variation.

A stepwise regression strategy was used to identify the most parsimonious model with optimal fit, balancing explanatory power and model simplicity. This approach allowed progressive testing of individual predictors (age, CRP, albumin, and CAR) and their interactions while avoiding model overfitting, which can occur easily in small datasets. Among all combinations, the interaction between age and CRP emerged as the key term improving model performance, suggesting that the diagnostic contribution of CRP was not uniform across age groups.

The significance of the age × CRP interaction indicates that younger dogs exhibited higher CRP levels and a greater likelihood of SIRS for equivalent CRP values compared with older animals. This finding does not imply that age itself is a protective factor but rather reflects a structural limitation of the study design, controls were significantly older than infected dogs. Consequently, the apparent age effect likely arises from the demographic imbalance between groups rather than a true biological modulation of risk. Still, the interaction term captures an important aspect of this bias, allowing the model to adjust for how CRP’s discriminative weight shifts with age.

From a diagnostic perspective, the final age × CRP model achieved excellent predictive accuracy, with an AUC of 0.93 and Cohen’s κ of 0.84, denoting substantial agreement with clinical classification. The optimal probability threshold determined by the ROC01 criterion (0.69) maximized overall classification accuracy, producing a positive predictive value of 0.97 and a negative predictive value of 0.85. In practice, this means that when the model estimates a probability of SIRS ≥ 0.69, the likelihood of true clinical SIRS is very high.

Nevertheless, these results should be interpreted cautiously. Statistical adjustment mitigates but cannot eliminate the influence of non–age-matched sampling. The model’s predictive power may partly reflect the underlying age distribution of the dataset rather than a biological interaction per se.

### 4.8. Limitations

Several limitations should be considered when interpreting the present findings. The sample size was relatively modest, and all parvovirus-positive dogs originated from a single referral hospital. This monocentric design may limit generalizability to broader clinical populations, particularly to primary-care or subclinical cases where disease severity and inflammatory magnitude are likely lower.

The control group was not age-matched to the parvoviral cohort because ethical regulations at our institution do not allow venipuncture in clinically healthy puppies unless medically indicated. Consequently, healthy controls could only be recruited among older dogs presenting for routine procedures. Although age-adjusted modelling was applied to mitigate this imbalance, complete elimination of age-related effects cannot be guaranteed. Therefore, the significant role of age in the final logistic regression model should be interpreted as a design limitation rather than as evidence of biological protection. Future studies should employ age-matched or stratified sampling to more accurately quantify the independent effect of CRP and CAR on systemic inflammation.

Another constraint relates to the cross-sectional sampling strategy: only one blood sample per dog was available within 24–48 h after hospital admission. This precluded assessment of temporal biomarker dynamics and may have attenuated associations with clinical SIRS status, as supportive therapy had typically already been initiated by that time. Serial sampling would enable better understanding of the kinetics and prognostic trajectories of CRP, albumin, and CAR throughout the course of disease.

Analytical limitations should also be acknowledged. The IDEXX Catalyst^®^ CRP assay’s upper quantification limit of 10.0 mg/dL may have underestimated true peak inflammatory responses in severely affected dogs, potentially leading to a ceiling effect in high-CRP cases.

Finally, the findings are specific to canine parvoviral enteritis and may not extrapolate to other infectious or inflammatory conditions without further validation. Larger, multicentric studies incorporating age-matched controls, broader disease contexts, and longitudinal biomarker profiling are warranted to confirm these results and refine clinically meaningful CAR and CRP thresholds.

## 5. Conclusions

CRP and CAR proved to be practical and complementary biomarkers for assessing systemic inflammation in dogs naturally infected with canine parvovirus. Both showed good diagnostic performance in identifying SIRS, with CRP serving as a rapid indicator of inflammatory activation and CAR providing a broader reflection of metabolic and nutritional imbalance. The optimal CRP threshold determined in this study (2.25 mg/dL) was slightly below the IDEXX positive cut-off (>3.0 mg/dL), suggesting that dogs within the manufacturer’s “mild or resolving inflammation” range (1.0–3.0 mg/dL) may already exhibit clinically relevant inflammatory changes. The inclusion of age in the regression models improved diagnostic precision but also highlighted the importance of demographic matching when evaluating biomarker performance.

Combined interpretation of CRP and CAR may assist clinicians in distinguishing dogs with mild, self-limited inflammation from those with severe systemic involvement requiring intensive care. Future multicentric and longitudinal studies should validate these findings in age-matched and heterogeneous populations, clarify biomarker kinetics during hospitalization, and explore integration with additional inflammatory markers to refine early detection and prognostic assessment of SIRS and sepsis in canine patients.

## Figures and Tables

**Table 1:** Demographic characteristics of parvovirus cases (PG) and controls (CG), and clinical profile of parvovirus cases.

	Groups		
Variable	Parvovirus (PG) N = 45 ^1^	Controls (CG) N = 15 ^1^	*p*-Value ^2^
Age (months)	2.00 (2.00–4.00)	12.00 (11.00–12.00)	<0.001
Sex:			0.084
Female	21 (47%)	11 (73%)	
Male	24 (53%)	4 (27%)	
Breed:			0.192
Purebred	29 (64%)	13 (87%)	
Mix breed	16 (36%)	2 (13%)	
CRP (mg/dL)	4.80 (2.80–9.10)	0.40 (0.20–0.40)	<0.001
Albumin (g/dL)	2.28 (1.89–2.65)	3.40 (3.06–3.73)	<0.001
CAR (CRP/Albumin ratio)	2.54 (1.28–3.58)	0.11 (0.06–0.13)	<0.001
Leukocytes (µ/L)	11 (6–17)	11 (9–14)	0.896
Temperature (°C)	38.30 (38.10–38.80)	38.50 (38.30–38.60)	0.688
Heart rate (bpm)	160 (144–184)		
Respiratory rate (rpm)	30 (24–36)		
Hospitalization time (days)	6 (5–8)		
Outcome: Discharged, n (%)	42 (93.3%)		
Outcome: Died, n (%)	3 (6.7%)		

^1^ Median (Q1–Q3); n (%); ^2^ Wilcoxon rank sum test; Fisher’s exact test.

**Table 2:** Spearman’s rank correlation coefficients (ρ) and 95% confidence intervals (CIs) between serum CRP, albumin, CRP/albumin ratio (CAR), and age in dogs (n = 60).

Variable 1	Variable 2	n	Spearman (ρ) [CI95%]	*p*-Value
CRP	Albumin	60	ρ = −0.53 [−0.69; −0.32]	<0.001
CRP	CAR	60	ρ = 0.96 [0.92; 0.98]	<0.001
CRP	Age (months)	60	ρ = −0.38 [−0.61; −0.09]	0.003
Albumin	CAR	60	ρ = −0.70 [−0.81; −0.53]	<0.001
Albumin	Age (months)	60	ρ = 0.54 [0.32; 0.72]	<0.001
CAR	Age (months)	60	ρ = −0.43 [−0.65; −0.15]	<0.001

**Table 3:** Agreement between clinical SIRS classification (Sykes) and IDEXX-based classification and diagnostic performance metrics.

(A) Cross-Tabulation (All Dogs, n = 60)			
Criterion	IDEXX: No SIRS	IDEXX: SIRS	Total
Sykes: No SIRS	21	5	26
Sykes: SIRS	8	26	34
Total	29	31	60
**(B) Diagnostic performance metrics (IDEXX vs. Sykes; 95% exact CIs)**			
Metric		Value (95% CI)	
Cohen’s κ		0.56	
Sensitivity (%)		76.5 (58.8–89.3)	
Specificity (%)		80.8 (60.6–93.4)	
Positive predictive value (%)		83.9	
Negative predictive value (%)		72.4	

## Data Availability

The original contributions presented in this study are included in the article/Supplementary Materials. Further inquiries can be directed to the corresponding author.

## Supplementary Materials

The following supporting information can be downloaded at: https://www.mdpi.com/article/10.3390/vetsci12121126/s1, Table S1: Criteria for diagnosis of Systemic Inflammatory Response Syndrome (SIRS) in dogs (adapted from Sykes); Table S2: Wilcoxon rank-sum test comparison of CRP, albumin, and CAR between SIRS-positive and SIRS-negative dogs (Sykes’ criteria); Table S3: Confusion matrix of the decision-tree classifier vs. SIRS (Sykes); Table S4: Diagnostic performance of the decision tree (exact 95% CIs); Table S5: Detailed model coefficients and performance metrics; Table S6: ROC01 thresholds and performance per logistic model.

## Abbreviations

The following abbreviations are used in this manuscript:

AIC	Akaike Information Criterion
APR	Acute-Phase Response
APP	Acute-Phase Proteins
AUC	Area Under the Curve
BICU	Biological Isolation and Containment Unit
CAR	C Reactive Protein/Albumin Ratio
CBC	Complete Blood Count
CI	Confidence Interval
CG	Control Group
CPV	Canine parvoviral enteritis
CRP	C-Reactive Protein
FVM-ULisboa	Faculty of Veterinary Medicine of University of Lisboa
HR	Heart Rate
HT	Hospitalization Duration
IQR	Interquartile Range
NPV	Negative Predictive Value
PCR	Polymerase Chain Reaction
PG	Parvovirus Group
PPV	Positive Predictive Value
ROC	Receiver Operating Characteristic
RR	Respiratory Rate
RT-qPCR	Real-Time Quantitative Polymerase Chain Reaction
SD	Standard Deviation
SIRS	Systemic Inflammatory Response Syndrome
TLC	Total Leukocyte Count
VIL	Virology and Immunology Laboratory
VTH	Veterinary Teaching Hospital
