# Supplementary material for: Predictive Prognosis Value of CRP Measurement and CAR in Dogs Infected with Parvovirus
Source: Vet Sci. 2025 Nov 27;12(12):1126. doi: 10.3390/vetsci12121126 (PMC12737769; doi:10.3390/vetsci12121126)
Supplement: Supplementary file 1 [file vetsci-12-01126-s001.zip › Table S4.DiagnosticPerformanceDecision.pdf]

Table S4. Diagnostic performance of the decision tree (exact 95% CIs).

Metric	Estimate	95% CI
Apparent prevalence	0.30	0.19–0.43
True prevalence	0.43	0.31–0.62
Sensitivity	0.69	0.48–0.86
Specificity	1.00	0.90–1.00
Positive predictive value	1.00	0.81–1.00
Negative predictive value	0.81	0.66–0.91
Positive likelihood ratio	Inf	NaN–Inf
Negative likelihood ratio	0.31	0.17–0.55
Correctly classified proportion (Accuracy)	0.87	0.75–0.94
