# Supplementary material for: Predictive Prognosis Value of CRP Measurement and CAR in Dogs Infected with Parvovirus
Source: Vet Sci. 2025 Nov 27;12(12):1126. doi: 10.3390/vetsci12121126 (PMC12737769; doi:10.3390/vetsci12121126)
Supplement: Supplementary file 1 [file vetsci-12-01126-s001.zip › TableS2_Tests_SIRS.pdf]

Table S2. *Wilcoxon rank-sum test comparison of CRP, albumin, and CAR between SIRS-positive and SIRS-negative dogs (Sykes' criteria).*

Biomarker	Statistic (W)	p-value
CRP (mg/dL)	111.500	<0.001
Albumin (g/dL)	669.500	0.001
CAR (CRP/Albumin)	120.000	<0.001
