# Supplementary material for: Predictive Prognosis Value of CRP Measurement and CAR in Dogs Infected with Parvovirus
Source: Vet Sci. 2025 Nov 27;12(12):1126. doi: 10.3390/vetsci12121126 (PMC12737769; doi:10.3390/vetsci12121126)
Supplement: Supplementary file 1 [file vetsci-12-01126-s001.zip › TableS3_Tree_Matrix.pdf]

Table S3. Confusion matrix of the decision-tree classifier vs. SIRS (Sykes).

Predicted	Actual: Negative	Actual: Positive	Total
Negative	18	0	18
Positive	8	34	42
