# Supplementary material for: Predictive Prognosis Value of CRP Measurement and CAR in Dogs Infected with Parvovirus
Source: Vet Sci. 2025 Nov 27;12(12):1126. doi: 10.3390/vetsci12121126 (PMC12737769; doi:10.3390/vetsci12121126)
Supplement: Supplementary file 1 [file vetsci-12-01126-s001.zip › TableS5-logistic_models.pdf]

Table S4. Detailed models coefficients and performance metrics

Models	Terms	Estimate	Std. Error	z value	p-valor
Model 1: age	Intercept	2.0647	0.5427	3.804	<0.001
Model 1: age	age	-0.3072	0.0839	-3.663	<0.001
Model 2a: age + CRP	Intercept	-0.1939	0.7493	-0.259	0.796
Model 2a: age + CRP	age	-0.2766	0.0921	-3.004	0.003
Model 2a: age + CRP	CRP	0.5253	0.1707	3.078	0.002
Model 2b: age + Albumin	Intercept	3.5098	1.3974	2.512	0.012
Model 2b: age + Albumin	age	-0.2601	0.0911	-2.854	0.004
Model 2b: age + Albumin	Albumin	-0.6732	0.5735	-1.174	0.240
Model 2c: age + CAR	Intercept	0.0455	0.7519	0.061	0.952
Model 2c: age + CAR	age	-0.2440	0.0890	-2.742	0.006
Model 2c: age + CAR	CAR	0.9331	0.3207	2.910	0.004
Model 3a: age + CRP + Albumin	Intercept	-0.5480	1.7673	-0.310	0.757
Model 3a: age + CRP + Albumin	age	-0.2842	0.0989	-2.873	0.004
Model 3a: age + CRP + Albumin	CRP	0.5378	0.1812	2.968	0.003
Model 3a: age + CRP + Albumin	Albumin	0.1441	0.6525	0.221	0.825
Model 3b: age + CRP + CAR	Intercept	-0.1365	0.7549	-0.181	0.856
Model 3b: age + CRP + CAR	age	-0.2916	0.0975	-2.990	0.003
Model 3b: age + CRP + CAR	CRP	0.7060	0.3668	1.925	0.054
Model 3b: age + CRP + CAR	CAR	-0.3759	0.6411	-0.586	0.558
Model 4: age * CRP	Intercept	2.6880	1.4648	1.835	0.066
Model 4: age * CRP	age	-1.0334	0.4412	-2.342	0.019
Model 4: age * CRP	CRP	0.0458	0.2410	0.190	0.849
Model 4: age * CRP	age:CRP	0.1021	0.0519	1.969	0.049

Models	AIC	logLik	Deviance	Null deviance	df resid	df null	n
Model 1: age	62.97	-29.49	58.97	82.11	58	59	60
Model 2a: age + CRP	49.14	-21.57	43.14	82.11	57	59	60
Model 2b: age + Albumin	63.56	-28.78	57.56	82.11	57	59	60
Model 2c: age + CAR	52.45	-23.23	46.45	82.11	57	59	60
Model 3a: age + CRP + Albumin	51.10	-21.55	43.10	82.11	56	59	60
Model 3b: age + CRP + CAR	50.84	-21.42	42.84	82.11	56	59	60
Model 4: age * CRP	43.82	-17.91	35.82	82.11	56	59	60
