# Supplementary material for: Predictive Prognosis Value of CRP Measurement and CAR in Dogs Infected with Parvovirus
Source: Vet Sci. 2025 Nov 27;12(12):1126. doi: 10.3390/vetsci12121126 (PMC12737769; doi:10.3390/vetsci12121126)
Supplement: Supplementary file 1 [file vetsci-12-01126-s001.zip › TableS6-ROC01_models_performance.pdf]

Table S5. ROC01 thresholds and performance per logistic model.

Model	AUC	ROC01 threshold	Sensitivity (ROC01)	Specificity (ROC01)
**Age**	0.87	0.728	0.85	0.85
**Age + CRP**	0.91	0.564	0.94	0.81
**Age × CRP**	0.93	0.694	0.97	0.85
