# Supplementary material for: Predictive Prognosis Value of CRP Measurement and CAR in Dogs Infected with Parvovirus
Source: Vet Sci. 2025 Nov 27;12(12):1126. doi: 10.3390/vetsci12121126 (PMC12737769; doi:10.3390/vetsci12121126)
Supplement: Supplementary file 1 [file vetsci-12-01126-s001.zip › Table_S1_Classification_SIRS.pdf]

Table S1. Criteria for diagnosis of Systemic Inflammatory Response Syndrome (SIRS) in dogs (adapted from Sykes)

**Parameters**	**Dog**
Temperature (°C)	< 37.8 or > 39.4
Heart rate (bpm)	> 140
Respiratory rate (rpm) / PCO_2	> 30 rpm or PCO_2 < 32 mmHg (venous or arterial)
Total leukocyte count	< 6,000 or > 16,000 cells/µL or > 3% band neutrophils
